# 

**Published:** 1850-09

**Authors:** 


BOOKS RECEIVED.

We are indebted to the courtesy of the publishers, and the polite attentions of Messrs. Beckwith & Morton, for the following books: *General Therapeutics and Materia Medica;* adapted for a Medical Text Book. By Robley Dunglison, M. D., &c. Fourth edition, revised and improved. Philadelphia: Lea & Blanchard. *Surgical Anatomy.* By Joseph Maclise, Surgion. With colored plates, 3d part. Lea & Blanchard. *Of the causes, nature, and treatment of Palsy and Apoplexy;* of the forms, seats, complications, and morbid relations of paralytic and apoplectic diseases. By James Copland, M. D., F. R. S. &c. Lea & Blanchard. *The Principles and Practice of Dental Surgery.* By Chapin A. Harris, M. D., D. D. S. Fourth edition, revised, modified, and greatly enlarged. With two hundred illustrations. Philadelphia: Lindsay & Blakiston. *Spectacles; their uses and abuses in long and short sightedness;* and the pathological conditions resulting from their irrational employment. By J. Sichel, M. D., &c. Translated from the French, by permission of the author, by Henry W. Williams, M. D., Fellow of the Massachusetts Medical Society, etc. Boston: Phillips, Sampson, & Co.

We shall endeavor to do justice to the merits of these various works, in the October number of this Journal. B.

### *Receipts of Medical Journal for Aug.* 1850.

| | |
|---|---|
| Dr. J. Smick, Athens, Ill. | $10 00 |
| J. Smith Woodsfield, Ohio, | 35 00 |
| R. Burwell, Buffalo, N. Y., | 10 00 |
| R. S. Newton, Cincinnati, Ohio, | 5 00 |
| G. M. Wharton, Tuscumbia, Ala., | 3 00 |
| J. A. Shuttlesworth, Campbellsville, Ky. | 18 40 |

